# Association between hours of work and subjective well-being. How do physicians compare to lawyers and accountants?

**DOI:** 10.1371/journal.pone.0295797

**Published:** 2023-12-15

**Authors:** Alberto Núñez-Elvira

**Affiliations:** Institute of Global Health Innovation (IGHI), Imperial College London, London, United Kingdom; Kwame Nkrumah University of Science and Technology, GHANA

## Abstract

Analyses of physician well-being typically rely on small and unrepresentative samples. In April 2011, the UK Office for National Statistics incorporated subjective well-being metrics (SWB) into the Annual Population Survey (APS), a well-established survey. This survey includes variables from the labor market, making APS an ideal source for measuring the association between work hours and SWB metrics and comparing among different professionals. Using APS data from 2011/12 to 2014/15, this study examined the association between SWB levels and work hours using multiple linear models for physicians (primary care physicians and hospital doctors), lawyers, and accountants. Of the 11,810 observations, physicians were more satisfied, happier, and less anxious; females were more stressed (10.7%); and age was negatively associated with happiness and satisfaction. Incorporating information on preferences to work more hours (underemployment) did not affect physicians’ but worsened the well-being of other professionals (lawyers and accountants). Surveyed physicians were less anxious, happier, and more satisfied than lawyers or accountants before Covid. Although the total work hours did not alter the SWB metrics, overtime hours for other professionals did. Increasing the working hours of underemployed physicians (with appropriate compensation) could be a relatively inexpensive solution to tackle the shortage of health workers in the short run.

## Introduction

Interest in incorporating well-being metrics into public policy decisions has grown over the last years [1]. These are usually classified as objective, subjective, or composite well-being metrics and help in the policymaking process. In a Report from the European Commission published in 2009, Stiglitz-Sen-Fitoussi proposed incorporating well-being metrics into national surveys to analyze quality of life within and between countries [2]. Using subjective well-being (SWB) metrics can be informative in the analysis of the health workforce because they provide important information about individuals’ true feelings and experiences when asked about their self-perceptions. This information is of high value for policymakers to inform and improve their design and evaluation of workforce planning policies [3].

In 2010, under the strategy Measuring National Well-being, the UK started gathering this information [4]. This UK strategy made metrics available in surveys owned by the Office for National Statistics (ONS). Information on subjective well-being (SWB) was surveyed using four questions (also known as ONS4) that ranked personal well-being on a 0–10 scale in four main dimensions: anxiety, happiness, life satisfaction, and worthiness of life. These four questions were first included in the Annual Population Survey (APS) in April 2011, the largest household survey in the UK. Therefore, APS represents the first official dataset in the UK that incorporates four SWB metrics (ONS4). Since then, these SWB questions have been added to other surveys such as the Wealth and Assets Survey, Living Costs and Food Survey, Crime Survey, and Opinions and Lifestyle Survey [5]. Nevertheless, other self-designed surveys also contain variables of well-being and work hours: the British Medical Association Quarterly Survey (BMAQs) series and the National General Practice Worklife Survey (NGPWS) [6–13].

Although there is a growing interest in understanding how physicians make their labor supply decisions, existing evidence usually relies on models that estimate the economic determinants of work hours [14, 15] or wages but leaves aside other variables that can influence those decisions. Understanding how physicians make their decisions on the main labor market outcomes can help tackle the existing shortage of physicians in many countries, offer solutions to the health workforce crisis, improve recruitment and retention policies, and identify potential problems. However, this is a difficult task for physicians in a context in which their wage rate is observed and associated with anticipated changes alongside their careers.

In the current context, the long-standing shortage of workers in the healthcare sector has grown interest in understanding other issues that impact labor market decisions and indirectly examining how labor market outcomes impact workers’ well-being. One of the main problems of discontent among physicians is hours of work [16], and much of this discontent comes from unpaid/paid overtime hours [17–20]. This discontent can be translated into stress [21–23], longer leaves [24], reduced productivity, or growing intentions to quit the profession [25]. Under the assumption that workers with lower well-being scores are more likely to drop out of the labor market [25–30], this study proposes an alternative method to approach physicians’ labor supply. Existing evidence indicates that working long hours may correlate positively with anxiety [17, 31, 32], and negatively with happiness and satisfaction [33] among physicians. Using SWB variables (anxiety, happiness, satisfaction, and depression), this study explored the appropriateness of using APS data to examine whether work hours are associated with subjective well-being (SWB) metrics included in the UK Annual Population Survey (APS) for physicians and compared them with other professionals. The APS also includes information on the main labor market outcomes and workers’ preferences to work more or fewer hours than the desired optimal level. Individuals who reported on the APS that they were working below their optimal level (underemployment) were more willing to work more hours at the current basic rate of pay, whereas those working above their optimal number of hours (overemployment) were more willing to reduce their work hours or were more likely to leave the profession.

The analysis relied on the APS data from 2011/12 to 2014/15. The reason for considering only these four years is to test the association between SWB variables and some labor outcomes, such as work hours in the early years when the SWB metrics were incorporated into APS, before their consideration in other surveys. By incorporating these metrics, APS offers the best source for exploring the relationship between SWB and labor market outcomes. The analysis also compares physician estimates with those of lawyers and accountants. Although the APS covers many occupations, this study restricted the comparator group to only two (lawyers and accountants) for simplicity. These professionals are considered a good comparator group in the private sector, following the guidelines of the Office of Manpower Economics (OME) in their pay comparison studies [34, 35]. Despite existing choices made by the OME, lawyers and accountants share certain characteristics with physicians. First, these three occupations require similar levels of university training, demanding a degree as the minimum requirement and postgraduate studies for specialization [36] and demand continuous professional development for career progression [37]. Moreover, they are strongly regulated by professional accreditation bodies that introduce regulations, such as licensing (physicians or lawyers) or accreditation (accountants) [38, 39]. These occupations have undergone an increasing participation of female workers in the last decades [40–42]. In addition, physicians, lawyers, and accountants usually report working long hours [43–46] compared to other professionals such as nurses or social workers [37]. This has raised concerns about gender discrimination in career progression and work-life balance as a result of feminization [47].

## Materials and methods

This study examines whether hours of work are associated with changes in SWB metrics (anxiety, happiness, satisfaction, or depression) included in the UK Annual Population Survey (APS) between 2011/12 and 2014/15 which offers an alternative and more comprehensive source than other relevant surveys from the BMA and the National GP Worklife Survey (NGWPS). The focus using the APS is to examine this association for physicians and other professionals (lawyers and accountants), using general linear regression models (ordinary least squares, OLS). This four-year period (2011/12–2014/15) was the first in which these SWB variables were available in the APS datasets. The main advantage of having this information in the APS is that it offers a good opportunity to examine whether the main labor market outcomes can be associated with changes in SWB variables.

The comparison between physicians and the other two occupations in the comparator group (lawyers and accountants) is supported by similarities in terms of education and career progression, feminization, and long work hours. This has been acknowledged by the Office of Manpower Economics (OME) in its well-known pay review reports. These similarities include shared criteria with physicians regarding entry requirements, training qualifications, intellectual rigor, career progression, pay scales [48], years of experience, career comparability, and pay data availability [49]. Hence, this study examined the suitability of APS for analyzing the relationship between work hours and SWB metrics to consider the convenience of using this APS data source for similar analyses.

The main regression model used to assess these associations is as follows:

$$W_{it}=d_{t}+\beta X_{it}+\delta O_{it}+\gamma L_{it}+\varepsilon_{it}$$

where ***W***_***it***_ is a vector of standardized SWB outcomes (anxiety, happiness, life satisfaction), ***d***_*t*_ is a vector of year dummies, ***X***_***it***_ is a set of sociodemographic characteristics, ***O***_***it***_ is a vector of occupations, and ***L***_***it***_ is a vector of labor market outcomes that includes hourly wages, different measures of hours of work (basic and overtime hours in main job, hours in second job and total hours of work in main and second job) and preferences to work more hours [50]. The availability of these labour market outcomes and sociodemographic variables enables to make different analyses by age, gender, or region. The SWB variables were standardized by subtracting the mean from each observation and dividing the result by the standard deviation of the distribution. These standardized variables were included as dependent variables in all the regression models. Some respondents reported their preferences to work more hours when they were asked whether they would like to work longer hours at the current basic rate of pay, given the opportunity. Those respondents were more likely to work part-time or below their optimal number of hours and were classified as workers in underemployment in the APS.

The analysis estimates different specification models, specifically five multiple linear regression models (OLS). The first two models (Models 1 and 2) control for female dummy, age, age-squared (age2), occupation in main job, underemployment dummy, and hours of work variable. We included respondents aged 26–70 years because physicians usually start their training as junior doctors at that age. The models provided in the Results section are described as follows: Model 1 includes an aggregated measure of hours; Model 2 breaks down total hours into basic and overtime hours in the main job and hours in the second job. The following specifications add hourly wages (in logs) as covariates (Models 3 and 4). Finally, Model 5 includes year dummies; ethnicity dummies (white, black, Asian, and other ethnicities); regional dummies (nine regions in England, Scotland, Wales, and Northern Ireland); and marital status dummies (married, divorced, separated, and widowed). The supporting information includes estimates from further specifications, conditioned by occupation.

### Data

Information on SWB has been reported regularly in the UK Labor Force Survey (LFS) documentation since 2011/12 and is available in the APS secure access version (SN7961, project number 98681). Both the LFS and APS are continuous household surveys and are representative of the characteristics of the UK population, with a large sample size of more than 300,000 respondents per quarter [51]. Both surveys are recommended for analyses that link labor market variables with other relevant variables. Both datasets were weighted to deliver information on the size and composition of the population by age and gender more accurately. The cross-sectional datasets of the APS gathers data every 13 weeks, and the outputs are delivered annually. This study compiled data from the APS and constructed a unique pooled cross-sectional dataset from 2011/1 to 2014/15 for the above mentioned purposes of this study.

APS contains SWB metrics and the most relevant variables from the labor market. Before April 2011, the well-being metrics were only available from the “APS Well-Being micro dataset”; however, since April 2011, they have been incorporated into the secure access APS datasets. The inclusion of SWB metrics and information from the LFS makes APS an excellent data source for comparisons on workforce topics [52] and represents a better data source than others such as the Understanding Society (UK) or the German Socio-Economic Panel (GSOEP). Answers to the SWB questions in the APS are ranked on an 11-point scale, ranging from 0 to 10, offering consistent scales and questions to the respondents. This consistency across answers facilitates clearer responses and helps in the analysis of the four questions separately [4]. Table 1 summarizes the main SWB questions in the APS.

**Table 1:** Subjective well-being (SWB) variables.

VARIABLE	SWB METRIC	QUESTION	Min	Max
**ANXIOUS**	Anxiety	*How anxious did you feel yesterday*? *(where nought is ‘not at all anxious’ and 10 is ‘completely anxious’)*;	0	10
**HAPPY**	Happiness	*How happy did you feel yesterday*? *(where nought is ‘not at all happy’ and 10 is ‘completely happy’)*;	0	10
**SATIS**	Life satisfaction	*Overall*, *how satisfied are you with your life nowadays*? *(where nought is ‘not at all satisfied’ and 10 is ‘completely satisfied’)*;	0	10
**WORTH**	Worthiness of life	*Overall*, *to what extent do you feel the things you do in your life are worthwhile*? *(where nought is ‘not at all worthwhile’ and 10 is ‘completely worthwhile’)*	0	10

Access to SWB datasets is granted through the UK Data Service, but access to the data owner requires users to comply with the requisites. First, users must hold an approved researcher status. Second, users must sign compulsory user agreements to comply with the data policy. This agreement was signed by the Director of Research and Innovation at Lancaster University when this study was conducted. No ethics committee was required for this study because it did not involve human participants.

The APS gathers information on relevant socioeconomic variables at the local level. The APS combines data from two of the five waves from the LFS with local data samples from England, Wales, and Scotland. Data were collected over 12 months and disseminated quarterly, including rich information on socioeconomic characteristics at the local level and variables that are more specific to the labor market (e.g., employment, unemployment, health, education, religion, and housing).

The APS sample is representative of the characteristics of the UK population and is larger than other surveys that include well-being metrics (see S1 Table), such as the Wealth and Assets Survey, Living Costs and Food Survey, Crime Survey, and Opinions and Lifestyle Survey [5]. Further checks were performed regularly to test the representativeness of this sample and to compare it with samples from administrative sources. In this study, the sample size was restricted to workers with a degree or above working as physicians (general practitioners [GPs] and hospital doctors) or in the two comparator groups proposed (lawyers and accountants). Occupation groups were identified using occupational classification (SOC2010). The breakdown into GPs and hospital doctors was obtained using industry classification codes (INDCM) following the LFS guidelines [53].

The total hours worked in the main job are an aggregated measure of the total usual hours in the main job (TTUSHR) and the actual hours in the second job (ACTHR2). Usual hours of work provide a better measure of usual working patterns on a regular basis, and are not affected by holidays, bank holidays, illnesses, or any other absence. Actual hours measure the number of hours that respondents work during the week and are seasonally adjusted to reflect absences from work [54, 55]. A further breakdown splits hours of work into basic usual hours (BUSHR), and overtime hours in the main job include both paid (POTHR) and unpaid (UOTHR) overtimes. Some variables reported that workers wanted to work more hours than usual at the current basic pay rate (UNDEMP), and, hence, they will be compensated for those additional hours. This can be understood as the concept of underemployment, in which respondents state their preference for working more hours at the current hourly wage. This information is included as a covariate in the regression model, as this can be associated with well-being levels. Hourly wages (HOURPAY) were reported by all respondents who reported earnings between £0 and £100 per hour in the week of the interview following the LFS guidelines [51]. This variable was transformed into logarithmic form to reduce the potential skewness in the distribution. The other covariates used were age, sex, marital status, ethnicity, country of birth, and regional and year dummy variables.

## Results and discussion

A total of 11,810 observations (904 general practitioners (GPs), 1,886 hospital doctors, 2,171 lawyers, and 6,849 accountants), working in the survey reference week were reported in the APS secure access dataset (SN7961) between 2011/12 and 2014/15. The sample included both salaried and self-employed workers. Earnings information is available only to salaried workers and accounts for 8,011 observations (245 GPs, 1,595 hospital doctors, 1,234 lawyers and 4,937 accountants). Table 2 summarizes the main descriptive statistics from the sample.

**Table 2:** Descriptive statistics.

	N	Mean	SD	Min	Max
Happiness	11810	7.46	2.16	0	10
Life satisfaction	11810	7.76	1.87	0	10
Anxiety	11810	3.05	2.74	0	10
General Practitioners (GPs)	904	0.11	0.31	0	1
Hospital doctors	1886	0.23	0.42	0	1
Lawyers	2171	0.25	0.43	0	1
Accountants	6849	0.41	0.49	0	1
Females	5403	0.46	0.50	0	1
Age	11810	42.79	10.49	26	70
Age2	11810	1940.53	945.72	676	4900
Hourly wage	8011	26.32	12.36	0	100
Hourly wage (logs)	8011	3.16	0.51	0	100
Employees (salaried workers)	8011	0.68	0.44	0	1
Total hours (main & 2nd)	11810	41.80	13.89	0	102
Basic usual hours (main job)	11810	37.76	12.38	0	97
Overtime hours (main job)	11810	3.56	5.89	0	55
Actual hours (2nd job)	11810	0.49	2.78	0	55
Want more hours (underemployment)	11810	0.04	0.19	0	1
More hours wanted	11810	0.04	0.19	0	1
Immigrant	11810	0.23	0.42	0	1
Single	11810	0.12	0.33	0	1
Married (co-living)	11810	0.82	0.38	0	1
Divorced	11810	0.03	0.18	0	1
Separated	11810	0.01	0.12	0	1
Widowed	11810	0.01	0.07	0	1
White	11810	0.80	0.40	0	1
Black	11810	0.03	0.16	0	1
Asian	11810	0.13	0.34	0	1
Other	11810	0.04	0.19	0	1
North East	11810	0.04	0.18	0	1
North West	11810	0.10	0.30	0	1
Yorkshire and Humberside	11810	0.08	0.26	0	1
East Midlands	11810	0.06	0.24	0	1
West Midlands	11810	0.07	0.25	0	1
Easter England	11810	0.06	0.33	0	1
London	11810	0.19	0.40	0	1
South East	11810	0.22	0.41	0	1
South West	11810	0.08	0.28	0	1
Wales	11810	0.04	0.19	0	1
Scotland	11810	0.07	0.26	0	1
Northern Ireland	11810	0.03	0.18	0	1
FY 2011/12	11810	0.24	0.43	0	1
FY 2012/13	11810	0.25	0.43	0	1
FY 2013/14	11810	0.26	0.44	0	1
FY 2014/15	11810	0.24	0.43	0	1

Table 3 summarizes the average total SWB scores and gender breakdown. The first column comprises all observations (pooled) without further breakdown. This enables researchers to compare how physicians’ average well-being levels changed in contrast to those of lawyers and accountants. The average anxiety level in the sample was lower for physicians than for lawyers or accountants. Among the physicians, GPs reported the lowest levels of anxiety. By gender breakdown, this pattern persisted. Most professionals worked more than 40 hours per week, with the exception of female GPs and accountants. The proportion of hospital doctors and accountants wanting to work more hours was above 5% in the sample, and 4% for GPs and lawyers.

**Table 3:** Average SWB measures and average work hours overall and by gender.

	POOLED				MALES				FEMALES			
	GPs	Hospital doctors	Lawyers	Accountants	GPs	Hospital doctors	Lawyers	Accountants	GPs	Hospital doctors	Lawyers	Accountants
**Anxiety**	2.59	2.81	3.44	3.06	2.32	2.68	3.25	2.96	2.81	2.97	3.61	3.20
**Happiness**	7.67	7.60	7.37	7.41	7.74	7.59	7.37	7.37	7.62	7.62	7.37	7.46
**Satisfaction**	7.96	7.94	7.65	7.70	7.98	7.91	7.63	7.67	7.94	7.98	7.68	7.74
**Worthiness**	8.54	8.45	7.85	7.79	8.49	8.38	7.75	7.73	8.58	8.54	7.93	7.87
**Total weekly hours worked**												
*Average*	37.73	46.00	42.74	40.05	43.30	48.16	45.91	42.15	33.21	43.31	39.90	37.20
**Underemployment—Preferences more hours**												
*Average age*	46.05	39.46	44.41	45.59	51.86	40.43	48.50	47.49	40.50	37.19	41.35	43.13
*Proportion workers*	0.04	0.05	0.04	0.06	0.03	0.07	0.05	0.06	0.05	0.03	0.03	0.05
*Average weekly hours worked*	26.3	43.28	31.5	34.16	29.52	45.27	34.01	36.62	24.7	37.95	28.35	30.01
*Number more hours wanted*	9.44	10.36	9.83	8.46	10.42	11.00	8.89	9.03	8.96	8.64	11.01	7.49

**Fig 1:**
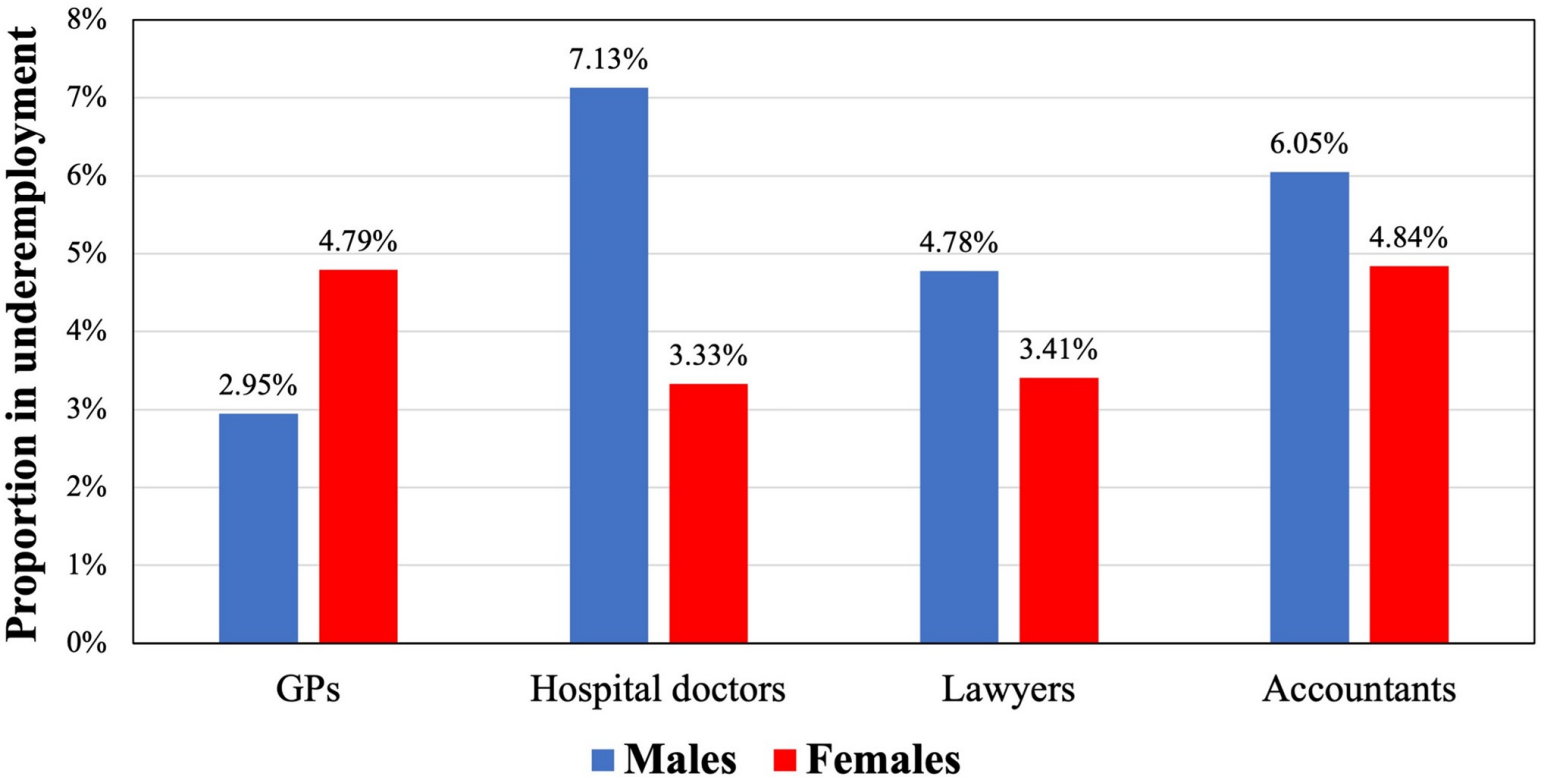
Proportion of workers working fewer hours than desired (underemployment).

Fig 1 shows the proportion of workers wanting to work more hours than at present by gender breakdown. When workers are not working their optimal hours (desired hours they would like to work), they are considered underemployed if their reported work hours are below their optimal (desired) level. This information is available in APS and is important because it enables us to identify whether respondents in underemployment would be willing to work at the current basic wage rate, and how many additional hours they would like to work at the current hourly wage rate. In Fig 1, male hospital doctors working more than 45 hours per week would like to work 11 more hours on average. GPs still report the lowest number of average work hours per week, with male GPs wanting to work nearly 10.5 more hours than at present and females wanting to work 9 more hours per week.

### Happiness

Table 4 provides regression estimates that include happiness as the dependent variable. Overall, physicians are happier than accountants are. Hospital doctors were the happiest of all occupations in all specifications. Overtime hours were negatively correlated with happiness (-1%). The estimates for females were not statistically significant. Adding hourly wages as a control in the regression model (Models 4.3–4.5) reports a positive association with happiness (4.9%) with the proposed hours’ breakdown into basic, overtime, and hours in the second job (Model 4.4). The inclusion of underemployment in the model as a covariate means a 7.5% drop in satisfaction (Model 4.2), which does not hold when controlling for hourly wages in the model. The coefficient for age shows a negative association with happiness (-2.9%) in the basic models (Models 4.1 and 4.2), without incorporating hourly wages as a covariate. Similar coefficients are found in Models 4.3–4.4 when hourly wages are included (-2.1%), showing a similar correlation between age and happiness, but it worsens (-0.4%) in the extended version (Model 4.5). The association between SWB and socioeconomic demographics such as immigration and marital status varies. In Model 4.5, immigrants are happier than native workers but at the 10% significance level. Married and divorced workers are happier than single workers are.

**Table 4:** Happiness estimates.

	4.1	4.2	4.3	4.4	4.5
**Female**	0.010	0.011	0.018	0.020	0.040*
	(0.020)	(0.020)	(0.023)	(0.024)	(0.024)
**Age**	-0.029***	-0.029***	-0.021***	-0.020**	-0.040***
	(0.006)	(0.006)	(0.008)	(0.008)	(0.008)
**Age2**	0.000***	0.000***	0.000**	0.000**	0.000***
	(0.000)	(0.000)	(0.000)	(0.000)	(0.000)
**Occupation (main job)**					
General practitioners	0.141***	0.128***	0.115*	0.094	0.076
	(0.035)	(0.035)	(0.066)	(0.066)	(0.066)
Hospital doctors	0.138***	0.137***	0.138***	0.128***	0.100***
	(0.026)	(0.027)	(0.030)	(0.030)	(0.032)
Lawyers	-0.016	-0.011	-0.031	-0.023	-0.019
	(0.025)	(0.025)	(0.032)	(0.032)	(0.032)
**Hourly wage (log)**			0.034	0.049**	0.034
			(0.023)	(0.024)	(0.025)
**Basic usual hours (main job)**		-0.004***		-0.004***	-0.003**
		(0.001)		(0.001)	(0.001)
**Overtime hours (main job)**		-0.011***		-0.012***	-0.011***
		(0.002)		(0.002)	(0.002)
**Actual hours (2nd job)**		0.003		0.004	0.004
		(0.002)		(0.003)	(0.003)
**Total hours (main & 2nd job)**	-0.004***		-0.006***		
	(0.001)		(0.001)		
**Underemployment**	-0.078*	-0.075*	-0.003	0.003	
	(0.042)	(0.042)	(0.051)	(0.051)	
**Immigrant**					0.060*
					(0.032)
**Married**					0.238***
					(0.028)
**Divorced**					0.152***
					(0.050)
**Separated**					-0.045
					(0.071)
**Widowed**					0.049
					(0.145)
**Constant**	0.770***	0.760***	0.547***	0.480***	0.768***
	(0.122)	-0.123	(0.167)	(0.171)	(0.176)
**Observations**	11810	11758	8011	7976	7970
**Year dummies**	*NO*	*NO*	*NO*	*NO*	*YES*
**Ethnicity dummies**	*NO*	*NO*	*NO*	*NO*	*YES*
**Regional dummies**	*NO*	*NO*	*NO*	*NO*	*YES*

Note: Standard errors in parentheses *** p<0.01, ** p<0.05, * p<0.10. Model 4.1 controls for gender, age, age2, occupation in main job dummies, underemployment dummy, and aggregated measure of total hours of work. Model 4.2 reproduces Model 4.1, but breaks down hours of work. Models 4.3 and 4.4 followed a similar rationale but added hourly wages (in logs) to the model. Model 4.5 adds year, ethnicity (white, black, Asian, and other), regional, and marital status dummies (married, divorced, separated, and widowed).

The models conditioned by occupation are provided in S2 Table. When estimates are significant, hours of work correlate negatively with well-being but at very low levels (less than 1%). The breakdown into basic, overtime, and hours in the second job shows that overtime only seems to affect the wellbeing of lawyers and accountants. Neither hourly wages nor being in underemployment seem to change well-being levels, except for hospital doctors, which are associated with greater happiness. However, there is a negative association between age and happiness. Socioeconomic demographics show that immigrant hospital doctors are less happy (-13%), but immigrant accountants are happier. Married workers were happier than single workers, and married GPs were the happiest among all occupations.

### Life satisfaction

Table 5 presents the coefficient regressions obtained from the models in which life satisfaction was the dependent variable. In the first two models (Models 5.1–5.2), physicians are more satisfied with their lives than accountants or lawyers. However, when the variable hourly wage is included as covariate, the coefficient for hospital doctors is significant and positive (17% in Models 5.3–5.4 and 12.6% in Model 5.5), showing that they are more satisfied with their lives than accountants. However, this is not the case for lawyers, who are less satisfied than accountants. The coefficient for general practitioners is not statistically significant for salaried GPs. Overtime is a source of dissatisfaction for all workers, but it does not mean a large source of discontent (approximately -1%). The other variables showed a greater correlation with satisfaction. Hourly wages show a positive association with satisfaction, averaging 10% SD (Models 5.3–5.5). Working fewer hours than desired (underemployment) was negatively correlated with life satisfaction in all the models. The addition of other socioeconomic demographics showed that married workers were more satisfied with their lives (30.3%) than singles, the comparator group, whereas separated and widowed workers were more dissatisfied with their lives (-23.6%).

**Table 5:** Life satisfaction estimates.

	5.1	5.2	5.3	5.4	5.5
**Female**	0.007	0.009	0.020	0.021	0.062***
	(0.020)	(0.020)	(0.022)	(0.023)	(0.022)
**Age**	-0.051***	-0.050***	-0.051***	-0.050***	-0.077***
	(0.006)	(0.006)	(0.008)	(0.008)	(0.008)
**Age2**	0.001***	0.001***	0.001***	0.000***	0.001***
	(0.000)	(0.000)	(0.000)	(0.000)	(0.000)
**Occupation (main job)**					
General practitioners	0.183***	0.168***	0.110*	0.086	0.052
	(0.035)	(0.035)	(0.063)	(0.063)	(0.062)
Hospital doctors	0.191***	0.192***	0.179***	0.176***	0.126***
	(0.026)	(0.026)	(0.028)	(0.029)	(0.030)
Lawyers	-0.039	-0.033	-0.081***	-0.073**	-0.076**
	(0.025)	(0.025)	(0.030)	(0.030)	(0.030)
**Hourly wage (log)**			0.095***	0.107***	0.109***
			(0.022)	(0.023)	(0.023)
**Basic usual hours (main job)**		-0.003***		-0.006***	-0.002*
		(0.001)		(0.001)	(0.001)
**Overtime hours (main job)**		-0.010***		-0.012***	-0.011***
		(0.002)		(0.002)	(0.002)
**Actual hours (2nd job)**		0.005**		0.004	0.005*
		(0.002)		(0.003)	(0.003)
**Total hours (main & 2nd job)**	-0.004***		-0.006***		
	(0.001)		(0.001)		
**Underemployment**	-0.251***	-0.251***	-0.202***	-0.200***	
	(0.042)	(0.042)	(0.049)	(0.049)	
**Immigrant**					0.049
					(0.030)
**Married**					0.303***
					(0.026)
**Divorced**					0.079*
					(0.047)
**Separated**					-0.236***
					(0.066)
**Widowed**					-0.330**
					(0.136)
**Constant**	1.307***	1.298***	1.132***	1.094***	1.320***
	(0.121)	-0.122	(0.159)	(0.162)	(0.166)
**Observations**	11810	11758	8011	7976	7970
**Year dummies**	*NO*	*NO*	*NO*	*NO*	*YES*
**Ethnicity dummies**	*NO*	*NO*	*NO*	*NO*	*YES*
**Regional dummies**	*NO*	*NO*	*NO*	*NO*	*YES*

Note: Standard errors in parentheses *** p<0.01, ** p<0.05, * p<0.10. Model 5.1 controls for gender, age, age2, occupation in main job dummies, underemployment dummy, and aggregated measure of total hours of work. Model 5.2 reproduces Model 5.1, but breaks down hours of work. Models 5.3 and 5.4 followed a similar rationale, but added hourly wages (in logs) to the model. Model 5.5 adds year, ethnicity (white, black, Asian, and other), regional and marital status dummies (married, divorced, separated, and widowed).

Conditioning models on occupation (S3 Table) reported that female GPs were less satisfied with their lives when all workers (including self-employed and salaried workers) were included in the sample. In these models (Models S3.1 and S3.2 in S3 Table), overtime hours seem to affect GPs more, but only by 2.1%. However, working fewer hours than desired (underemployment) is negatively correlated with GPs, lawyers, and accountants. The inclusion of hourly wages positively affects accountants or lawyers (a 1 per cent change in hourly wages will increase their satisfaction by 9–12% for lawyers and 13–16% for accountants). The coefficients for overtime hours are twofold for lawyers compared with accountants. Underemployment seems to be associated with lower levels of satisfaction only for salaried lawyers and accountants but not for physicians.

### Anxiety

Table 6 summarizes the regression estimates from the models in which anxiety is the dependent variable. Females were more anxious than males in all models, ranging from 10% to nearly 13%. Age is positively associated with anxiety in the basic specification (Models 6.1–6.2). When hourly wages are incorporated into the regression model, this association disappears (Models 6.3–6.5). Regression coefficients show that hospital doctors are approximately 13% less anxious than accountants, but lawyers are more anxious than accountants (12% in Models 6.1–6.2 and between 15% and 16% in Models 6.3–6.5). Overtime hours correlated positively with anxiety levels (1%), whereas hourly wages were negatively associated with anxiety levels, showing that a 1 per cent increase in hourly wages would mean a reduction in anxiety by 4.7% in Model 6.4, and -5.9% in Model 6.5. Working fewer hours than desired (underemployment) increased anxiety in most models in most models, but at the 10% significance level, except in Model 6.3, where the coefficient was significant at the 5% significance level.

**Table 6:** Anxiety estimates.

	6.1	6.2	6.3	6.4	6.5
**Female**	0.127***	0.129***	0.107***	0.105***	0.100***
	(0.020)	(0.020)	(0.023)	(0.024)	(0.024)
**Age**	0.015***	0.015***	0.008	0.008	0.013
	(0.006)	(0.006)	(0.008)	(0.008)	(0.008)
**Age2**	-0.000***	-0.000***	-0.000	-0.000	-0.000
	(0.000)	(0.000)	(0.000)	(0.000)	(0.000)
**Occupation (main job)**					
General practitioners	-0.189***	-0.176***	-0.060	-0.043	-0.046
	(0.035)	(0.035)	(0.066)	(0.066)	(0.067)
Hospital doctors	-0.129***	-0.135***	-0.132***	-0.124***	-0.124***
	(0.026)	(0.026)	(0.030)	(0.030)	(0.033)
Lawyers	0.127***	0.123***	0.164***	0.155***	0.154***
	(0.025)	(0.025)	(0.032)	(0.032)	(0.032)
**Hourly wage (log)**			-0.028	-0.047*	-0.059**
			(0.024)	(0.024)	(0.025)
**Basic usual hours (main job)**		0.003***		0.003**	0.002*
		(0.001)		(0.001)	(0.001)
**Overtime hours (main job)**		0.011***		0.013***	0.013***
		(0.002)		(0.002)	(0.002)
**Actual hours (2nd job)**		0.000		-0.001	-0.001
		(0.002)		(0.003)	(0.003)
**Total hours (main & 2nd job)**	0.004***		0.005***		
	(0.001)		(0.001)		
**Underemployment**	0.080*	0.077*	0.106**	0.096*	
	(0.042)	(0.042)	(0.051)	(0.052)	
**Immigrant**					-0.006
					(0.033)
**Married**					-0.019
					(0.028)
**Divorced**					-0.062
					(0.050)
**Separated**					-0.012
					(0.071)
**Widowed**					0.154
					(0.146)
**Constant**	-0.506***	-0.495***	-0.339**	-0.246	-0.238
	(0.122)	-0.122	(0.168)	(0.172)	(0.178)
**Observations**	11810	11758	8011	7976	7970
**Year dummies**	*NO*	*NO*	*NO*	*NO*	*YES*
**Ethnicity dummies**	*NO*	*NO*	*NO*	*NO*	*YES*
**Regional dummies**	*NO*	*NO*	*NO*	*NO*	*YES*

Note: Standard errors in parentheses *** p<0.01, ** p<0.05, * p<0.10. Model 6.1 controls for gender, age, age2, occupation in main job dummies, underemployment dummy, and aggregated measure of total hours of work. Model 6.2 reproduces Model 6.1, but breaks down hours of work. Models 6.3 and 6.4 followed a similar rationale but added hourly wages (in logs) to the model. Model 6.5 adds year, ethnicity (white, black, Asian, and other), regional and marital status dummies (married, divorced, separated, and widowed).

In the conditioned models in the S4 Table, females were more anxious than males were. In the models that included all respondents, female GPs were the most anxious (models S4.1 and S4.2 in S4 Table), whereas in models that only considered salaried workers, female lawyers showed the largest statistically significant coefficient. The coefficient for female salaried GPs was not statistically significant. Hourly wages were negatively correlated with accountants’ anxiety levels. The coefficients for physicians were not statistically significant. Overtime hours are associated with increasing anxiety for lawyers and accountants, but the increase is small (about 2% and 1%, respectively). Underemployment is associated with greater anxiety among salaried accountants.

## Discussion

The interest in understanding physicians’ well-being responds to the longstanding concern of health systems that report suffering from a shortage of health workers. The existing premise that stressed workers are more likely to drop out of the market justifies the analysis of work hours and SWB metrics to identify potential risks that may exacerbate the need to recruit and motivate health workers and physicians. In England, two surveys have been used to analyze burnout and well-being among physicians: the British Medical Association Quarterly Survey (BMAQs) series and the National General Practice Worklife Survey (NGPWS). However, they rely on their own questionnaires, which are difficult to access, and focus solely on physicians. The BMAQs reported a growing proportion of physicians with decreasing morale and satisfaction [7–10], with General Practitioners (GPs) reporting the lowest morale and satisfaction and identifying potential risks that could harm the retention of physicians and exacerbate the shortage of those professionals. The NGPWS also reported low levels of job satisfaction among physicians since 2010, with hours of work and remuneration being the main sources of dissatisfaction [56–58]. This study examined the APS as an alternative data source to the BMAQ and NGPWS. Considering only physicians, this study has shown that APS is a reliable data source for analyzing the association between work hours and subjective well-being levels, and complements other existing databases with the inclusion of more variables that permit the comparison of other labor market outcomes sources, such as underemployment, and compare physicians with lawyers and accountants.

For simplicity, the analysis restricts the comparator groups to these two occupations (lawyers and accountants). The choice of these occupations in the control group relies on the similarities of these professionals with physicians in the increasing feminization in those professions, career progression, access to practice or working conditions. These criteria are summarized in areas such as education level, licensing, career progression, long work hours, and pay. Physicians need to undertake undergraduate and postgraduate studies before starting their specialization as junior doctors.

Existing literature on subjective well-being signals an important association between work hours and well-being levels. Although this relationship has been tested in working environments [59], it remains unclear whether working long hours can impact the quality of life. For example, some studies found that working long hours worsened subjective well-being metrics [59] through higher burnout levels and lower satisfaction [60], whereas others reported a positive association between work hours and job satisfaction [61], especially overtime hours. This positive association can be justified from a psychological perspective in which individuals link working overtime hours with self-esteem and a growing perception of necessity in the job. This study contributes to narrowing this gap in the literature on physicians’ well-being by analyzing the association between SWB metrics and work hours.

A generally accepted conclusion is that work hours are associated with higher anxiety levels, depression, stress or burnout [17–20]. However, the existing evidence is inconclusive when examining the relationship between work hours and SWB, especially for hospital doctors. Some studies have found that growing burnout negatively affects physicians’ well-being [21, 22, 62] and has been linked to increasing dropouts for depression [63] and anxiety [23, 31, 64–67]. Thus, the more hours physicians worked, the less satisfied with their lives and the more anxious they were. When physicians are less happy and more anxious, this can reduce their performance, decrease their productivity and the quality of care delivered, but increase their likelihood of quitting. In the UK, physicians have reported working long hours and heavy workloads.

Heavy workloads and long working hours have been associated with lower happiness and life satisfaction [68], increased anxiety, and consequently, growing job stress [63], occupational stress [62], burnout [69], and sick leave [24]. However, most studies have failed to compare them with those of other professionals. The pre-Covid pandemic estimates obtained in the present analysis showed that hospital doctors and GPs were less anxious than accountants and lawyers (Table 6). The pandemic might have had an impact on physicians’ SWB well-being, but this requires further analysis using different estimation models and is beyond the scope of the present study. In the conditioned models (S4 Table), work hours did not seem to be associated with greater anxiety among physicians. However, the results for lawyers and accountants showed a positive correlation, resulting in increased anxiety levels, although the coefficient was modest. These findings contradict the existing evidence that corroborates the association between working long hours and increasing levels of anxiety, stress, and job dissatisfaction [32, 57]. This can be controversial for physicians who complain about their growing levels of burnout and stress [6–13], which are especially reported by newly qualified physicians [70].

Further breakdown into basic and overtime hours in the main job and hours in the second job is more informative than considering the variable that compiles basic, overtime, and hours in the second job (total hours in the main and second jobs). Hours in the second job were not associated with changes in well-being levels in any of the metrics except for anxiety (see S4 Table) for accountants, but in a small proportion. Van der Hulst et al (2001) showed that when overtime exceeded the regular daily work hours (8 hours), workers were more likely to suffer from burnout especially those working 12 hours or more [19]. Nonetheless, they considered only full-time workers, and both full-time and part-time workers agreed to overtime. It seems tempting to make such a breakdown in the analysis; however, this was not the aim of the present study, and will not add much to the analysis. For example, this is relevant for some health professionals such as salaried GPs or temporary workers (locums), who can decide the number of hours they work or are hired to cover a vacancy for temporary absence or within a specific number of weeks, respectively.

One of the most interesting results comes from the inclusion of the underemployment variable as a covariate in the analysis. This covariate has not been included in previous studies. Underemployment refers to workers who work below their optimal number of hours but want to work more hours than at present at the current basic hourly wage. This provides information on whether respondents maximize their utility at their current number of work hours. This could be useful for workforce planners and policymakers to identify potential workers who would be willing to work more hours, increasing the total number of hours worked by health workers, such as physicians. Workers under this definition of unemployment usually have worse levels of well-being than those who work their optimal number of hours [71]. The analysis covered respondents aged 26–70 who had a degree in their field. Two types of workers were identified in this study as underemployed workers. The first group comprised young workers [72]. Second, part-time workers usually work fewer than 30 hours per week [73]. The latter usually report lower levels of well-being [74]. In this study, underemployment was negatively correlated with life satisfaction (Table 5) and positively correlated with anxiety (Table 6). In the conditioned models, underemployment was negatively correlated with well-being for lawyers and accountants, but not for physicians (S2 Table), and negatively correlated with anxiety levels for the same professionals (S3 Table). The results for the physicians were not statistically significant. The identification of this subgroup of workers, for example, among physicians, could be an interesting way to address the perceived shortage of work hours among health workers. With this information, policymakers could consider expanding the number of compensated work hours of physicians working fewer hours than desired to reach their optimal level. Not only might this increase their well-being, but it could also be an easy and relatively inexpensive way to increase the total number of work hours supplied by physicians. This approach may help to address the shortage of physicians in the short run.

Another interesting relationship to consider is the link between wages and work hours, which has been widely explored [75–77]. Most studies associate income with higher levels of happiness and satisfaction [78], lower anxiety [50], and increased well-being, particularly among low-income workers. In this study, when earnings variables are included as covariates, such as hourly wages, the coefficients corroborate the findings from existing evidence that higher wages increase happiness and satisfaction, and decrease anxiety (Tables 4–6). Further analyses provided in the Supporting Information also confirm this positive association between well-being and hourly wages, with results from existing evidence on the retention of the physicians´ workforce, suggesting that higher wages increase the retention of health workers [79, 80]. However, the analysis of life satisfaction reported noteworthy coefficients for GPs (S3 Table) when the models controlled for hourly wages. This may mean that salaried GPs value more other variables that the models do not capture and might have a greater impact on satisfaction.

Further consideration is the inclusion of salaried and self-employed workers in this study. Johnson et al [65] compared salaried workers (employees) among 26 occupations in the UK using a general questionnaire in occupational stress and found that physicians are worse off than accountants, ranking lower on psychological well-being (17.82 vs 17.47) and job satisfaction (25.66 vs 18.74). Controlling for hourly wages from the APS, this study focused solely on salaried workers in the models in which hourly wages were included as covariates. The main findings reported different conclusions from those of Johnson et al., showing that physicians were better off than accountants and lawyers were. The analysis could have included a dummy variable to distinguish between salaried and self-employed workers; however, earnings variables are not available in the APS for the self-employed, and would have limited other detailed analyses. refrained from including such a variable, because it did not add much to the present analysis for various reasons. First, the proportion of self-employed workers varies among occupations. Although the proportion of self-employed hospital doctors is small in the UK, approximately 70% of GPs are self-employed in primary care services, and are known as GP partners. These GPs bill their services to the National Health System (NHS) and the NHS pays them back for their services. Second, the absence of the earnings variable for self-employed workers limits other analyses and there is heterogeneity in the proportion of self-employed GPs compared to the other occupations considered. Approximately 70% of UK GPs are self-employed (known as partner GPs) compared to 29% of lawyers, 25% of accountants, and only 2% of hospital doctors. Therefore, the analysis covered both self-employed and salaried GPs and provided further breakdown only for salaried models as supplementary material.

This is informative because it enables to infer how hours of work or other relevant variables are associated with salaried workers’ well-being. In the analysis developed here, the estimates suggest that physicians are happier, more satisfied with their lives, and less anxious than other workers do. The inclusion of all workers is relevant for two main reasons. First, self-employed workers are happier than salaried workers because they have greater autonomy [81]. However, the existing evidence is inconclusive, and other studies focusing on salaried UK GPs reported that salaried workers are as satisfied as the self-employed, more satisfied with their wages and weekly hours, but more stressed than the self-employed [82]. Second, salaried GPs account for 30–35% of the total GP headcount, but the proportion of female workers is greater than that in other occupations.

Satisfaction is an important issue for physicians´ well-being. Numerous studies have investigated physicians´ job satisfaction [33, 83–85]; however, most analyses rely on self-collected data from their own questionnaires and there is little evidence of overall satisfaction using SWB variables. Overall, physicians were satisfied with their jobs (85.7% physicians)., as reported by Joyce et al. (2011). Van Ham et al. surveyed the evidence on the job satisfaction of GPs [33] and concluded that low income, long work hours, administrative burden, and heavy workloads could worsen satisfaction. This could be relevant for physicians. If physicians are happy and satisfied with their jobs, they are less likely to quit. In this study, coefficients showed that physicians are more satisfied with their lives than accountants or lawyers (Table 5, Models 5.1–5.2), but results for GPs were not significant (Models 5.3–5.5). This non-statistical significance of hours of work on SWB, controlling for hourly wages, complicates the conclusion that salaried physicians are more satisfied than accountants, as corroborated by the analyses of the full sample. As the data analyzed pre-dated the COVID pandemic, physicians and other professional SWB measures should be reassessed in a future study, given the stress physicians experience on the frontline of care delivery during the pandemic.

Additional considerations in this analysis relate to the growing feminization of the medical profession. Feminization is an important issue in the medical profession [42, 86, 87]. Occupations with a greater proportion of female workers are characterized by a large proportion working part-time [87], and hours of work tend to be lower compared to other occupations. In the context of the study discussed here, estimates show that females are more anxious than males (Table 6), overall, and more satisfied (Table 5, Model 5.5). However, conditioned models (S2 Table) reported that female GPs were unhappier and less satisfied with their lives (Models S2.1 and S2.2 in S2 Table). Models that included both self-employed and salaried workers also reported higher anxiety levels among the female GPs. The higher levels of anxiety among female workers and their magnitudes are consistent with the existing literature and within the estimated range [50].

This study examined the association between work hours and SWB to test the convenience of using APS for such analyses. In fact, it includes other work-related variables in the labor market that may correlate with well-being. Contrary to the findings of some studies, the results of the present study indicate that physicians are, on average, less anxious, happier, and more satisfied with their lives than lawyers or accountants during the four-year period of analysis. This is supported by models that exclude hourly wages as a covariate. Including hourly wages as a covariate, most estimates for physicians are not significant. However, the conclusions of this study need to be interpreted with caution, as no causal relationship was reported in this study.

This study has some limitations. Hourly wages in a second job are not included as covariates. The proportion of physicians with a second job was small and did not significantly affect the results. Income variables for self-employed workers were not reported in the survey, which could have made it possible to test whether self-employed workers are happier than salaried workers [81] with more accurate results. Working conditions can impact SWB metrics [88]. In APS, working conditions are reported with variables such as basic work hours, overtime hours, size of the workplace, or distance to work. If these variables are included, it is important to control for as many working condition variables as possible to ascertain potential sources of dissatisfaction that may lead to stress and depression in the work environment. This analysis refrained from including some of these variables and restricted the analysis to exploring the association of SWB metrics with work hours, breaking down work hours into basic, overtime, and second job hours. This study aimed to test whether there are associations between work hours and SWB metrics using the APS to report correlations between covariates and whether SWB metrics and causality should be explored. One reason for including only the relevant variables is that SWB metrics do not focus solely on work satisfaction or anxiety. Finally, the subgroup analysis performed conditioning on occupations did not explore potential biases from the groups in further detail. Further subgroup analyses should incorporate differential item functioning (DIF) analyses to reduce biases and the possibility that there are superior or inferior groups [89].

Future research should expand on these analyses for the following reasons. First, more evidence on workforce retention is needed, showing either associations or causal relationships. The literature on nurse retention, but not that of physicians, is growing. Health workers who were satisfied and happy were more likely to remain in their roles. Second, these are predictors of potential discontent; therefore, they are a good metric for identifying problems in the early stages. Finally, this analysis can be reproduced in different contexts. One of these contexts could be the application of similar studies on labor supply in the context of the covid-19 pandemic. Other studies should consider examining the main determinants of labor supply over the life cycle or evaluating the impact of workers who would like to reduce their current hours of work for less pay (overemployed workers) for different reasons.

## Conclusions

APS is useful for testing whether there is an association between SWB variables and hours of work, wages, and underemployment among different occupations. The main finding of this study contradicted what has been said so far, and found that physicians were, on average, less anxious than lawyers and accountants with similar entry requirements, training qualifications, intellectual rigor, career comparability, and pay scales. Furthermore, physicians were happier and more satisfied than other professionals in the comparison group. The association between hours of work and well-being metrics was modest when we controlled for the aggregate measure of total hours in main and secondary jobs. The inclusion of hourly wages reported an expected positive association with happiness and satisfaction, which is supported by the existing evidence. Nonetheless, this was not the case for salaried GPs in models conditioned by occupation. The additional breakdown into basic, overtime, and second job hours also reported a modest correlation with overtime work with the expected sign (positive or negative). The inclusion of a variable that reported information on fewer hours worked compared to the respondents’ optimal hours was informative in identifying a potential source of discontent or stress.

## Supporting information

S1 Table SWB (ONS4) datasets. (PDF)

S2 Table Happiness estimates (conditioned models). (PDF)

S3 Table Life satisfaction estimates (conditioned models). (PDF)

S4 Table Anxiety estimates (conditioned models). (PDF)
