# Supplementary material for: Association between hours of work and subjective well-being. How do physicians compare to lawyers and accountants?
Source: PLoS One. 2023 Dec 15;18(12):e0295797. doi: 10.1371/journal.pone.0295797 (PMC10723739; doi:10.1371/journal.pone.0295797)
Supplement: S1 Table — (PDF) [file pone.0295797.s001.pdf]

S 1 Table. SWB (ONS4) datasets

Organisation	Survey	Topics covered	First asked	Frequency of update
Office for National Statistics (ONS)	Annual Population Survey (APS)	Labour market data including employment and unemployment, as well as housing, ethnicity, religion, health and education.	April 2011 to March 2012	Annual
	Wealth and Assets Survey	Level of assets, savings and debt; saving for retirement; how wealth is distributed among households or individuals; and factors that affect financial planning.	July 2011 to June 2012 (Wave 3)	Bi-annual
	Living Costs and Foods Survey	Household spending patterns for the consumer prices index and for GDP figures and detailed information on food consumption and nutrition.	April 2011 to March 2012	Annual
	Crime Survey for England and Wales	Experience of crime and attitudes to crime-related issues such as the police, the criminal justice system, and perceptions of crime and anti-social behaviour.	April 2012	Annual with quarterly updates
	Opinions and Lifestyle Survey	Collects information on a variety of topics that are too small to have surveys of their own. Topics that have been previously commissioned include smoking habits, cancer awareness, charitable giving, climate change and disability.	April 2011	Monthly
University of Oxford and ONS	Time Use Survey	Diary entry survey. The substantive domains are main activity (49 categories), secondary activity (10 categories), location and means of transport (11 categories) and with whom (8 categories). The temporal identifier holds information on the time when episodes start and end.	April 2014 to March 2015	Annual
Cabinet Office	National Citizenship Services Evaluation	Social mixing; transition to adulthood; teamwork, communication and leadership; and community involvement.	2014	Not updated
	Youth Social Action Survey	Social Action (Only Satisfaction and Worthwhile included).	2014	Annual
Department for Work and Pensions (DWP)	Life Opportunities Survey	Measures how disabled and non-disabled people participate in society in a number of areas which include:<br>• work<br>• education<br>• social participation	2013 to 2014	Not updated
	The National Study of Work Search and Wellbeing Findings	Psychological health and well-being of Jobseekers Allowance (JSA) claimants.	2011	Not updated
	English Longitudinal Study of Ageing (ELSA)	Information on the health, social, wellbeing and economic circumstances of the English population aged 50 years and older.	April 2012 to March 2013	Annual
Department of Health	What about YOUth? Survey	Young people's health, diet, what they do in their free time, bullying and whether they smoke, take drugs or drink alcohol.	2014	Not updated
Ministry of Defence (MoD)	Armed Forces Continuous Attitude Survey (AFCAS)	Information on the views and experiences of MoD personnel which helps shape policies for training, support, and the terms and conditions of service.	2012	Annual

Department for Business, Energy and Industrial Strategy (BEIS)	Families Continuous Attitude Survey (FAMCAS)	Information on personals in the MoD spouses in a number of areas including accommodation, healthcare, education and childcare, and deployment.	2012	Annual
	Impact of FE learning Survey	Attitudes towards further education, including funding, readiness of information, guidance, and decision making process.	2012	Not updated
Department for Communities and Local Government (DCLG)	English Housing Survey	Age, type, condition and energy efficiency of housing stock and the characteristics of households.	2013 to 2014	Annual
The Department for Digital, Culture, Media & Sport (DCMS)	Taking Part Survey	Participation in and engagement with cultural and sporting activities at the individual level, and pathways in and out of participation and engagement.	2013 to 2014	Annual
	Community Life Survey	Volunteering, charitable giving, local action and networks and well-being.	2013 to 2014	Annual
Foods Standards Agency	Food and You	Reported behaviours, attitudes and knowledge relating to food issues such as reported food purchasing, storage, preparation and consumption. It also looks at eating habits, influences on where respondents choose to eat out and experiences of food poisoning.	2014	Bi-annual
Welsh Government	The National Survey for Wales	Opinions on a wide range of issues affecting people living in Wales and their local area.	April 2012 to March 2013	Annual
Central Statistics Office Ireland	Quarterly National Households Survey	Labour force estimates that include the official measure of employment and unemployment in the state (International Labour Organisation (ILO) basis).	2013	Well-being module not updated
Natural England	Monitor of Engagement with the Natural Environment (MENE): The Natural Survey on People and the Natural Environment	How people use the natural environment, includes the:<br>• type of destination<br>• duration<br>• mode of transport<br>• distance travelled<br>• expenditure<br>• main activities<br>• motivations<br>• barriers to visiting	2012 to 2013	Annual
UK Civil Service	Civil Service People Survey	Civil service staff attitudes and experiences of work.		
Sainsbury's, Oxford Economics and National Centre for Social Research	Living Well Index	What does it mean to live well? How well are we really living as a nation, and why? This study aims to provide the answers – by defining, measuring and tracking, over a number of years, what it means to live well in Britain.	2012	Annual
Higher Education Statistics Agency	Measuring Graduate Subjective Well-being Outcomes through Destination of Leavers from Higher Education (DLHE)	The survey which will gather insightful and comprehensive information about graduate outcomes. The four ONS personal well-being questions are optional.	2017	Annual

One Parent Families Scotland and Scottish Poverty and Inequality Research Unit at Glasgow Caledonian University	Single Parents Community Connections Survey	Aims to be the largest ever survey of single parents in Scotland. The results will feed into OPFS and GCU's Community Connections project funded by the Scottish Government Innovation fund. The project aims to tackle isolation, loneliness and poor mental health among single parents.	2018	Not updated
The Land Trust	Perceptions Survey and Social Value Study	The Land Trust is dedicated to providing free public open space for the benefit of communities. Land Trust commissioned Carney Green to undertake a Social value assessment of its sites.	2015	Not updated
Natural Resources Wales	People Survey 2015	Our People Survey was carried out in order to gauge honest opinions from staff on how they feel about working for Natural Resources Wales.	2016	Not updated
Active Lives Survey	Sport England	Measuring the number of people aged 14 and over taking part in sport and physical activity.	2015	Annual
Centre for Regional Economic and Social Research (CRESR) and Institute for Employment Research (IER), University of Warwick	Active Lives Survey - Children and Young People Survey	Includes 3 of the ONS 4 - does not include the anxiety question.	2017	Annual
	Big Lottery Talent Match Survey	An evaluation survey of the initial entrants onto the Talent Match programme. The overall objectives of the programme are to support 25,000 individuals with the goal of 5,400 entering employment.	2014	Not updated
Isle of Man Government	Health and Lifestyle Survey 2017	The areas of interest for this survey were:<br>• general health<br>• diet and physical activity<br>• smoking<br>• alcohol and drug consumption<br>• well-being	2016	Annual
Higher Education Policy Institute	Student Academic Experience Survey	The survey investigates the learning and teaching experiences of students, including satisfaction with courses, reasons for dissatisfaction, experience of different-sized classes, total time spent working, perceptions of value-for-money, institutional spending priorities and a focus on student well-being.	2014	Annual
