# Supplementary material for: Association between hours of work and subjective well-being. How do physicians compare to lawyers and accountants?
Source: PLoS One. 2023 Dec 15;18(12):e0295797. doi: 10.1371/journal.pone.0295797 (PMC10723739; doi:10.1371/journal.pone.0295797)
Supplement: S3 Table — (PDF) [file pone.0295797.s003.pdf]

S 3 Table. Life satisfaction estimates (conditioned models)

	S3.1				S3.2				S3.3				S3.4				S3.5			
	GPs	Hospital doctors	Lawyers	Accountants	GPs	Hospital doctors	Lawyers	Accountants	GPs	Hospital doctors	Lawyers	Accountants	GPs	Hospital doctors	Lawyers	Accountants	GPs	Hospital doctors	Lawyers	Accountants
**Female**	-0.164**	0.018	-0.002	0.028	-0.155**	0.032	0.024	0.024	0.162	0.055	-0.042	0.031	0.122	0.06	-0.012	0.022	0.052	0.042	0.025	0.067**
	(0.076)	(0.045)	(0.048)	(0.025)	(0.077)	(0.047)	(0.049)	(0.026)	(0.159)	(0.049)	(0.059)	(0.028)	(0.162)	(0.050)	(0.060)	(0.029)	(0.173)	(0.051)	(0.059)	(0.028)
**Age**	-0.058***	-0.042***	-0.086***	-0.043***	-0.058***	-0.044***	-0.087***	-0.041***	-0.009	-0.049***	-0.091***	-0.039***	-0.02	-0.050***	-0.093***	-0.037***	-0.024	-0.056***	-0.125***	-0.064***
	(0.020)	(0.013)	(0.013)	(0.007)	(0.020)	(0.014)	(0.013)	(0.007)	(0.047)	(0.016)	(0.018)	(0.010)	(0.048)	(0.017)	(0.018)	(0.010)	(0.052)	-0.018	-0.019	-0.01
**Age2**	0.001**	0.000***	0.001***	0.000***	0.000**	0.000***	0.001***	0.000***	0.000	0.001***	0.001***	0.000***	0.000	0.001***	0.001***	0.000***	0.000	0.001***	0.001***	0.001***
	(0.000)	(0.000)	(0.000)	(0.000)	(0.000)	(0.000)	(0.000)	(0.000)	(0.001)	(0.000)	(0.000)	(0.000)	(0.001)	(0.000)	(0.000)	(0.000)	(0.001)	(0.000)	(0.000)	(0.000)
**Hourly wage (log)**									-0.385***	0.055	0.089	0.133***	-0.432***	0.052	0.109*	0.153***	-0.505***	0.019	0.123**	0.160***
									(0.139)	(0.057)	(0.056)	(0.028)	(0.142)	(0.059)	(0.057)	(0.029)	(0.148)	(0.060)	(0.060)	(0.029)
**Basic usual hours (main job)**					-0.007**	0.002	-0.003	-0.004***					-0.007	-0.002	-0.007**	-0.008***	-0.005	-0.001	-0.004	-0.004**
					(0.003)	(0.002)	(0.002)	(0.001)					(0.006)	(0.003)	(0.003)	(0.002)	(0.006)	(0.003)	(0.003)	(0.002)
**Overtime hours (main job)**					-0.021**	-0.006*	-0.017***	-0.009***					0.021	-0.005	-0.024***	-0.013***	0.021	-0.005	-0.022***	-0.012***
					(0.009)	(0.003)	(0.003)	(0.002)					(0.018)	(0.004)	(0.004)	(0.002)	(0.020)	(0.004)	(0.004)	(0.002)
**Actual hours (2nd job)**					0.002	0.004	0.008	0.004					0.01	0.001	0.003	0.006*	0.021	0.001	0.005	0.007**
					(0.009)	(0.005)	(0.006)	(0.003)					(0.014)	(0.006)	(0.010)	(0.003)	(0.015)	(0.006)	(0.010)	(0.003)
**Total hours (main & 2nd job)**	-0.007***	-0.001	-0.004***	-0.004***					-0.002	-0.003	-0.011***	-0.007***								
	(0.002)	(0.002)	(0.002)	(0.001)					(0.005)	(0.002)	(0.002)	(0.001)								
**Underemployment**	-0.370**	-0.018	-0.315***	-0.292***	-0.364**	-0.014	-0.303***	-0.293***	-0.081	-0.02	-0.346**	-0.246***	-0.113	-0.021	-0.316**	-0.246***				
	(0.171)	(0.095)	(0.116)	(0.052)	(0.171)	(0.095)	(0.116)	(0.052)	(0.261)	(0.098)	(0.146)	(0.062)	(0.263)	(0.099)	(0.146)	(0.062)				
**Immigrant**																	0.099	-0.125**	0.128	0.103**
																	(0.211)	(0.060)	(0.087)	(0.040)
**Married**																	0.348**	0.150**	0.309***	0.336***
																	(0.165)	(0.063)	(0.064)	(0.032)
**Divorced**																	-0.063	0.053	0.253**	0.042
																	(0.360)	(0.128)	(0.117)	(0.057)
**Separated**																	-0.373	0.189	-0.487***	-0.287***
																	(0.297)	(0.164)	(0.184)	(0.083)
**Widowed**																	0.368		0.512	-0.413***
																	(0.955)		(0.430)	(0.146)
**Constant**	2.074***	1.115***	2.125***	1.116***	2.047***	1.067***	2.135***	1.104***	1.691	1.150***	2.247***	0.795***	2.175*	1.145***	2.128***	0.754***	2.437**	1.228***	2.453***	0.925***
	(0.469)	(0.289)	(0.290)	(0.162)	(0.469)	(0.296)	(0.289)	(0.162)	(1.083)	(0.337)	(0.376)	(0.210)	(1.120)	(0.351)	(0.380)	(0.216)	(1.165)	(0.372)	(0.391)	(0.219)

**Observations**	904	1,886	2,171	6,849	903	1,871	2,161	6,823	245	1,595	1,234	4,937	245	1,586	1,228	4,917	245	1,585	1,227	4,913
**Year dummies**	*NO*	*NO*	*NO*	*NO*	*NO*	*NO*	*NO*	*NO*	*NO*	*NO*	*NO*	*NO*	*NO*	*NO*	*NO*	*NO*	*YES*	*YES*	*YES*	*YES*
**Ethnicity dummies**	*NO*	*NO*	*NO*	*NO*	*NO*	*NO*	*NO*	*NO*	*NO*	*NO*	*NO*	*NO*	*NO*	*NO*	*NO*	*NO*	*YES*	*YES*	*YES*	*YES*
**Regional dummies**	*NO*	*NO*	*NO*	*NO*	*NO*	*NO*	*NO*	*NO*	*NO*	*NO*	*NO*	*NO*	*NO*	*NO*	*NO*	*NO*	*YES*	*YES*	*YES*	*YES*

*Note: Standard errors in parentheses *** p<0.01, ** p<0.05, * p<0.10*
